# Karyotypes, B-chromosomes and meiotic abnormalities in 13 populations of *Alebra albostriella* and *A. wahlbergi* (Hemiptera, Auchenorrhyncha, Cicadellidae) from Greece

**DOI:** 10.3897/CompCytogen.v7i4.6411

**Published:** 2013-11-26

**Authors:** Valentina G. Kuznetsova, Natalia V. Golub, Dora Aguin-Pombo

**Affiliations:** 1 Department of Karyosystematics, Zoological Institute, Saint Petersburg, 199034, Russia; 2 University of Madeira, 9000-390 Funchal, Madeira, Portugal; 3 Centro de Investigaçao en Biodiversidade e Recursos Genéticos (CIBIO), Vairão, Portugal

**Keywords:** Karyotype, meiosis, chromosomal associations, translocations, macrospermatids, B-chromosomes, Greek populations, *Alebra*, Cicadellidae, Auchenorrhyncha

## Abstract

In this work 13 populations of the leafhopper species *Alebra albostriella* (Fallén, 1826) (6 populations) and *A. wahlbergi* (Boheman, 1845) (7 populations) (Cicadellidae: Typhlocybinae) from Greece were studied cytogenetically. We examined chromosomal complements and meiosis in 41 males of *A. albostriella* sampled from *Castanea sativa*, *Fagus sylvatica* and *Quercus cerris* and in 21 males of *A. wahlbergi* sampled from *C. sativa*, *Acer opalus* and *Ulmus* sp. The species were shown to share 2n = 22 + X(0) and male meiosis of the chiasmate preductional type typical for Auchenorrhyncha. In all populations of *A. albostriella* and in all but two populations of *A. wahlbergi* B chromosomes and/or different meiotic abnormalities including the end-to-end non-homologous chromosomal associations, translocation chains, univalents, anaphasic laggards besides aberrant sperms were encountered. This study represents the first chromosomal record for the genus *Alebra* and one of the few population-cytogenetic studies in the Auchenorrhyncha.

## Introduction

The leafhopper genus *Alebra* Fieber, 1872 (Cicadellidae: Typhlocybinae) comprises a complex of phytophagous species with several degrees of association to deciduous trees and shrubs. This Holarctic genus is represented in Europe by six valid species and several host associated populations of unknown taxonomic status. The taxonomy of *Alebra* is difficult due to the very slight morphological differences in male genital structures, a significant degree of intraspecific color pattern variation and the common occurrence of two or more species on the same food plant (Drosopoulos and Loukas 1988, Aguin-Pombo 2002). In the family Cicadellidae 387 species in 263 genera have been studied in respect to karyotype (Kuznetsova and Aguin-Pombo in press) but until now the genus *Alebra* remained totally untouched by chromosomal investigation.

Chromosomal polymorphisms in natural populations may play a significant role in speciation (White 1978, King 1993, Kawakami et al. 2011). This can easily be proven in cases in which chromosome rearrangements are easily detected as in some dipterans having giant polytene chromosomes in salivary glands. Quite the opposite situation is found in the Auchenorrhyncha with their rather small holokinetic chromosomes. Due to the absence of localized centromeres, the identification of rearrangements in holokinetic chromosomes is either difficult (e.g. translocations and deletions) or even completely impossible (duplications and inversions) if routine chromosome staining is applied. Despite the fact that approximately 820 auchenorrhynchan species have so far been karyotyped, all the data obtained concern almost exclusively chromosome numbers and gross karyotype morphology, and only few records of chromosomal polymorphisms have been published (for review see Kuznetsova and Aguin-Pombo in press).

In the present work, cytogenetic analysis of *Alebra albostriella* (Fallén, 1826) and *Alebra wahlbergi* (Boheman, 1845) was performed using routine chromosome staining. A study of 13 Greek populations of these species inhabiting different deciduous trees was undertaken to reveal whether the populations of these species display any polymorphism for chromosomal complements and meiotic patterns.

## Materials and methods

**Figure 1.**
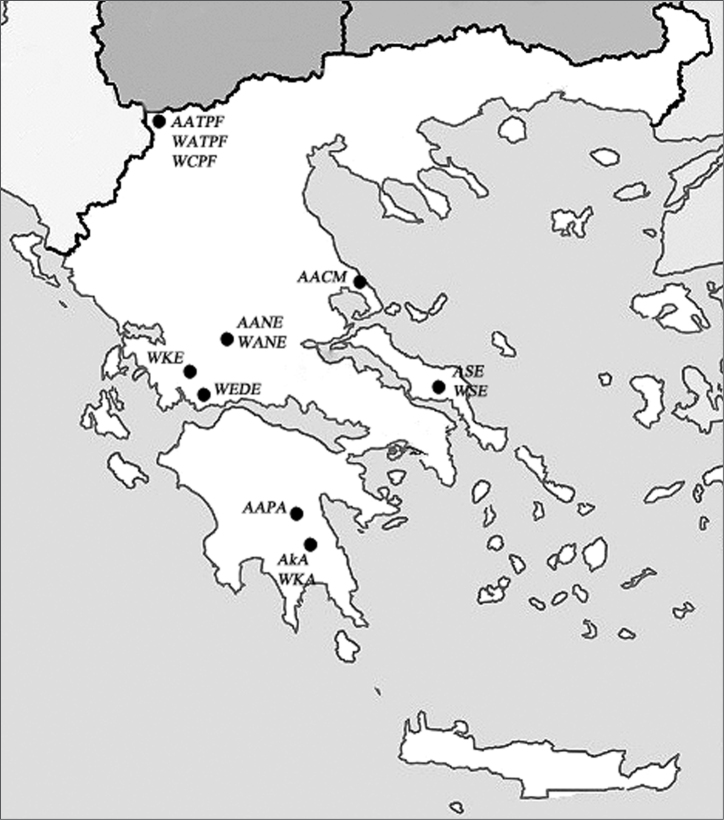
Map showing the collection localities of *Alebra albostriella* and *Alebra wahlbergi* in Greece.

Altogether, 41 males from 6 populations of *Alebra albostriella* and 21 males from 7 populations of *Alebra wahlbergi* inhabiting 5 different species of deciduous trees in Greece have been collected from 1989 to 1992 on plant foliage with a sweeping net. The locality names, altitude, data of collection and food plants are listed in Table 1, and the places of collection are also mapped on Fig. 1. For chromosome studies, adult males were fixed in Carnoy solution (3:1 ethanol and glacial acetic acid) and stored at -10°C. Chromosomal analysis was performed using conventional squashing procedure. Testes were dissected out, stained with 2% acetic orcein and squashed in a drop of 45% acetic acid under an 18-mm square coverslip. From 1 to 14 individuals in each population were examined. Chromosome preparations were analyzed under a Leica DM 4000B microscope (Leica Microsystems Wetzlar GmbH, Germany) with a 100× objective. Images were taken with a Leica DFC 350 FX camera using Leica Application Suite 2.8.1 software with an Image Overlay module. The data obtained are presented in Tables 2–4.

**Table 1.** Studied material.

Species	Population code	Locality	Altitude above sea level	Food plant	Data of collection	Number of studied males
*Alebra albostriella*	ASE*	Steni-Euboea Il.	440 m	*Castanea sativa*	8–9.07.1990	10
	AKA	Kastanitsa-Arkadia	850 m	*Castanea sativa*	25.06.1990<br/> 10.08.1989	2<br/> 3
	AACM	Anilio-Chania-Magnisia	990 m	*Castanea sativa*	23.07.1990	5
	AAPA	Agios Petros-Arkadia	990 m	*Castanea sativa*	15–16.7.1990	4
	AANE	Agios Nicolaos-Eurytania	1000 m	*Castanea sativa*	01.08.1991	2
	AATPF**	Agia Triada-Prespes-Florina	1200 m	*Fagus sylvatica*	14–21.08.1990	14
				*Quercus cerris*	20.08.1990	1
*Alebra wahlbergi*	WEDE	Evinos Delta-Etoloakarnania	20 m	*Ulmus* sp.	25.06.1991	5
	WSE	Steni-Euboea Il.	440 m	*Castanea sativa*	8–9.07.1990	2
	WKE	Kerasovo-Etoloakarnania	520 m	*Acer opalus*	14.06.1992	4
	WKA	Kastanitsa-Arkadia	850 m	*Castanea sativa*	25.06.1990	1
	WANE	Agios Nicolaos-Eurytania	1020 m	*Castanea sativa*	01.08.1991	3
	WCPF	Caries-Prespes-Florina	1100 m	*Acer opalus*	19.08.1990	1
	WATPF	Agia Triada-Prespes-Florina	1200 m	*Acer opalus*	14–21.08.1990	5

*Here and elsewhere we use abbreviations to refer to different populations of a species.

** Specimens of *Alebra albostriella* from the AATPF locality represent two different populations one occurring on *Fagus sylvatica* and the other on *Quercus cerris* (see Aguin-Pombo 2002 for details).

**Table 2.** B-chromosomes, meiotic abnormalities and macrospermatids in *Alebra albostriella*.

Populations (N=6)	Food plants	Males No (N=41)	Number of B-chromosomes	Meiotic abnormalities and macrospermatids
ASE	*Castanea sativa*	1	0	univalents
		2	0	end-to-end non-homologous associations anaphasic laggards macrospermatids
		3	0	end-to-end non-homologous associations macrospermatids
		4	0	end-to-end non-homologous associations macrospermatids
		5	0	anaphasic laggards macrospermatids
		6	0	macrospermatids
		7	0	macrospermatids
		8	0	macrospermatids
		9	0	-
		10	2	-
AKA	*Castanea sativa*	1	0	univalents
		2	0	-
		3	0	-
		4	0	-
		5	0	-
AACM	*Castanea sativa*	1	1	anaphasic laggards macrospermatids
		2	0	macrospermatids
		3	0	macrospermatids
		4	0	macrospermatids
		5	0	-
AAPA	*Castanea sativa*	1	0	macrospermatids
		2	0	-
		3	0	-
		4	0	-
AANE	*Castanea sativa*	1	1	end-to-end non-homologous associations univalents anaphasic laggards
		2	0	end-to-end non-homologous associations anaphasic laggards macrospermatids
AATPF	*Fagus sylvatica*	1	1	macrospermatids
		2	0	univalents macrospermatids
		3	0	end-to-end non-homologous associations
		4	0	macrospermatids
		5	0	macrospermatids
		6	0	macrospermatids
		7	0	macrospermatids
		8	0	macrospermatids
		9	0	macrospermatids
		10	0	macrospermatids
		11	0	macrospermatids
		12	0	macrospermatids
		13	0	macrospermatids
		14	0	-
	*Quercus cerris*	15	0	anaphasic laggards

**Table 3.** B-chromosomes, meiotic abnormalities and macrospermatids in *Alebra wahlbergi*.

Populations (N=7)	Food plants	Males No (N=21)	Number of B-chromosomes	Meiotic abnormalities and macrospermatids
WEDE	*Ulmus* sp.	1	2	univalents
		2	0	end-to-end non-homologous associations macrospermatids
		3	0	macrospermatids
		4	0	-
		5	0	-
WSE	*Castanea sativa*	1	0	univalents
		2	0	end-to-end non-homologous associations
WKE	*Acer opalus*	1	2	macrospermatids
		2	0	univalents
		3	1	-
		4	0	-
WKA	*Castanea sativa*	1	0	-
WANE	*Castanea sativa*	1	0	end-to-end non-homologous associations multiple translocation chains
		2	0	end-to-end non-homologous associations
		3	0	univalents
WCPF	*Acer opalus*	1	0	-
WATPF	*Acer opalus*	1	0	macrospermatids
		2	0	-
		3	0	-
		4	0	-
		5	0	-

**Table 4.** Frequency of B chromosomes in *Alebra albostriella* and *Alebra wahlbergi*.

Male N=7	Number of B chromosomes per cell	Total number of MI studied	Number of MI with B chromosomes	Frequency of B chromosomes per individual, %
***Alebra albostriella***				
1-AACM	1	460	3	0,65
1-AANE	1	370	4	1,08
10-ASE	2	98	82	83,7
1-AATPF	1	112	2	1,8
***Alebra wahlbergi***				
1-WEDE	2	28	17	60,7
1-WKE	2	107	84	78,5
3-WKE	1	180	2	1,1

## Results

### Alebra albostriella

#### Standard karyotype and meiosis

**Figure 2.**
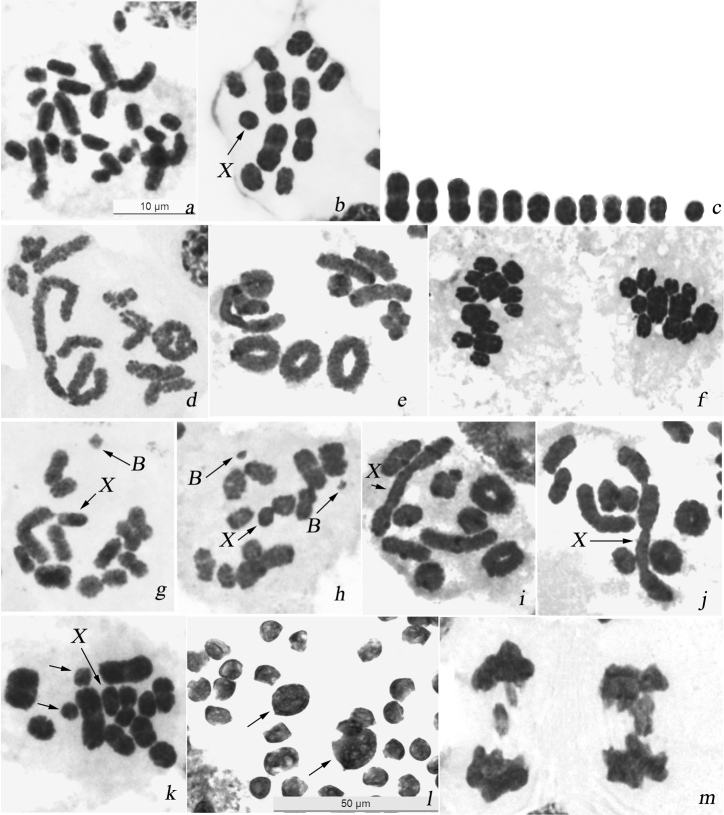
Karyotype and male meiosis in *Alebra albostriella*: **a** Mitotic metaphase showing 23 chromosomes **b** MI showing 11 bivalents and univalent X **c** karyogram prepared from MI (b) **d** diakinesis showing bivalents with one terminal/subterminal chiasma and a bivalent with two subterminal chiasmata **e** diakinesis showing bivalents with one terminal/subterminal chiasma, a bivalent with interstitial chiasma and 4 ring bivalents each with two terminal/subterminal chiasmata **f** two daughter AI with n=11 and n=12, respectively **g** diakinesis showing one B chromosome **h** diakinesis showing two B chromosomes **i** diakinesis with end-to-end association of two bivalents and X **j** diakinesis with end-to-endassociation ofthree bivalents and X **k** MI with one medium-sized bivalent as univalents (arrowed) **l** macrospermatids of different size (arrowed) among normal spermatids **m** AI with lagging chromosomes. Bar = 50 µm in **l** and 10 µm in other figures.

In males, the majority of cells showed 23 chromosomes at mitotic metaphases (Fig. 2a) and 12 units at meiotic metaphases I (MI) (Fig. 2b, c). The karyotype is asymmetric with two size groups of chromosomes. In mitosis six larger chromosomes and other chromosomes constituting a decreasing series in size were present. Chromosomes had no primary constrictions, i.e. centromeres and the sex chromosome could not be identified. At MI, 11 autosomal bivalents, including three larger, and a univalent X-chromosome were encountered (n=11 + X). Male karyotype formula of the species is thus as follows: 2n=22 + X(0). The univalent X-chromosome was similar in size to one of the larger half-bivalents within the group of smaller chromosomes and its location at MI was random. Bivalents mainly had a single terminal/subterminal or, rarer, interstitial chiasma, however in few nuclei up to four rings were present indicative of two terminal/subterminal chiasmata being formed in the larger bivalents (Fig. 2d, e). At anaphase I (AI), all the autosomes segregated to opposite poles and the X moved to one pole without dividing. The reductional division resulted thus in two daughter metaphase II (MII) cells with 11A+X and 11A, respectively (Fig. 2f).

#### B-chromosomes

In 4 out of 20 males analyzed in the populations ASE, AACM, AANE (sampled from *Castanea sativa*) and AATPF (sampled from *Fagus sylvatica*) one or two small B-chromosomes (additional to the standard complement) were found (Table 2). In the polymorphic populations, three males (1-AACM, 1-AANE and 1-AATPF) showed a single B-chromosome with the frequency of about 1% per specimen while male 10-ASE had a pair of B-chromosomes in about 80% of MI (Table 4). B-chromosomes were different in size in different males while always appreciably smaller than the X-univalent and negatively heteropycnotic at late prophase and MI (Fig. 2g, h). At MI, B-chromosome(s) showed random distribution relative to autosomal bivalents and X-chromosome. In the case of two B-chromosomes, they did not show any connection to each other (Fig. 2h).

#### Meiotic abnormalities

Different kinds of meiotic abnormalities were encountered in 12 males (29% of the total number of males) sampled from all the 6 populations (Table 2). In males 2, 3 and 4 of ASE (from *Castanea sativa*), in both studied males of AANE (from *Castanea sativa*), and in male 3 of AATPF (from *Fagus sylvatica*) two to four bivalents were occasionally associated by ends. The univalent X-chromosome was very often involved in these associations. Non-homologous telomeres did not touch intimately each other but unstained gaps were seen between the bivalents (Fig. 2i, j). In some males (Table 2), one or two middle-sized bivalents were seen as univalents in some cells at diakinesis and MI (Fig. 2k). In addition, populations ASE, AACM, AANE and AAPA (from *Castanea sativa*) and AATPF (from *Fagus sylvatica*) the majority of studied males (61%; N=25) showed macrospermatids coexisting with normal spermatids within a cyst. Macrospermatids were different in size being either approximately twice larger or much larger than the normal spermatids (Fig. 2l). Some males with aberrant spermatids displayed also one or other type of meiotic abnormalities, including lagging chromosomes at anaphases (Fig. 2m) (Table 2).

### Alebra wahlbergi

#### Standard karyotype and male meiosis

**Figure 3.**
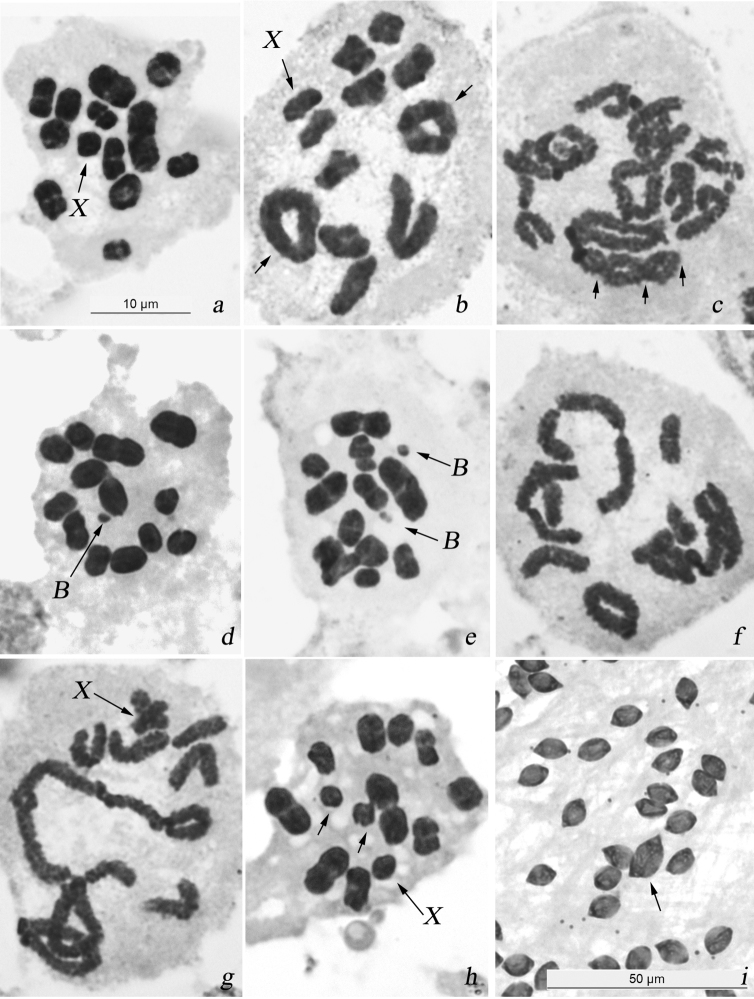
Karyotype and male meiosis in *Alebra wahlbergi*: **a** MI showing 11 bivalents and univalent X **b** diakinesis showing bivalents with one terminal/subterminal chiasma, a bivalent with interstitial chiasma and 2 ring bivalents each with two terminal/subterminal chiasmata **c** diplotene/diakinesis showing a bivalent with three (at least) chiasmata (arrowed) **d** MI with one B chromosome **e** MI with two B chromosomes **f** diakinesis with end-to-end association of three bivalents **g** diplotene/diakinesis showing translocation chain involving 4 bivalents **h** MI with one medium-sized bivalent as univalents (arrowed) **i** macrospermatid (arrowed) among normal spermatids. Bar = 50 µm in **i** and 10 µm in other figures.

In sampled males, 11 autosomal bivalents and the univalent X-chromosome were present in cells at diakinesis and MI (Fig. 3a, b), male karyotype formula of this species being thus 2n = 22 + X(0). Much as in *Alebra albostriella*, this karyotype was asymmetric with three larger bivalents and 8 smaller bivalents, the X-chromosome being similar in size to one of the larger half-bivalents in this second group. The chromosomes had no centromeres. The univalent X-chromosome was located randomly at diakinesis and MI. The bivalents mainly had a single terminal/subterminal or rarely interstitial chiasma, however, two chiasmata (rings on Fig. 3b) and occasionally three chiasmata (arrowed on Fig. 3c) could be formed in larger bivalents. In few cells at diakinesis and metaphase I four bivalents or (in male 1-WNE) even six bivalents with two chiasmata each were observed (not shown). Both autosomes and X-chromosome separated reductionally during the first division and divided equationally during the second division of meiosis.

#### B-chromosomes

In 3 out of 9 males analysed in the populations WEDE (sampled from *Ulmus* sp.) and WKE (sampled from *Acer opalus*) one or two B-chromosomes were encountered (Table 3). The polymorphic male 3-WKE showed a single B-chromosome in about 1% of MI (Fig. 3d, Table 4). Males 1-WEDE and 1-WKE showed each a pair of Bs at MI with frequencies of approximately 60% and 80%, respectively (Fig. 3e, Table 4). In every case B-chromosomes were very small, negatively heteropycnotic and distributed randomly with reference to each other, to the bivalents and to the X-chromosome.

#### Meiotic abnormalities

Meiotic abnormalities were encountered in 11 males (52% of the total number of males) sampled from 5 out of 7 studied populations. Populations WKA and WCPF showed no meiotic disturbances however in our study they were represented by only one male each (Table 3). In males 2-WEDE, 2-WSE, 1- and 2-WANE the bivalents occasionally formed associations involving two or three bivalents connected by telomere ends. Non-homologous telomeres did not touch intimately each other but unstained gaps were seen between the bivalents (Fig. 3f). In addition, male 1-WANE had nuclei at diakinesis with X-chromosome, 7 bivalents and a translocation chain of four bivalents united by chiasmata (Figs 3g). The chromosomal complement of these cells was in fact n = 7AA + 1(4AA) + X. Unfortunately, because of poor spreading of chromosomes in the slide no statistical analysis of the occurrence of translocation chains in this male was possible. Further still, we failed to detect the number of bivalents involved into certain translocation chains. In males 1-WEDE, 1-WSE, 2-WKE and 3-WANE, one of the middle-sized bivalents was present as univalents at MI (Fig. 3h). In populations WEDE, WKE and WATPF, 4 out of 14 males showed macrospermatids which were of approximately twice the normal size and coexisted with normal spermatids within a cyst (Fig. 3i). Among males with macrospermatids, only that 2-WEDE displayed meiotic disturbances namely the end-to-end non-homologous associations of bivalents (Table 3).

## Discussion

### Standard karyotypes and meiosis

*Alebra albostriella* and *Alebra wahlbergi* were found to have the same karyotype of 2n = 22 + X(0) encountered without variation in 30,6% of studied males (N=19) and with some variation due to polymorphism in the rest of males (N=43) . The species share likewise a similar gross morphology of karyotypes. Both karyotypes are asymmetric in terms of the heterogeneity of chromosome size: one size group includes three pairs of larger chromosomes and the other group includes 8 pairs of smaller chromosomes. Within every group, chromosomes represent continuous gradation in size and therefore can not been reliably distinguished by conventional cytogenetic approaches. The X-chromosome is close by size to one of the larger chromosomes within the smaller-sized group. Chromosomes are holokinetic as in other Hemiptera; that is, the centromeric activity is dispersed along the length of each chromosome rather than concentrated at one point (Schrader 1935).

In spite of holokinetic nature of chromosomes, there are only a few Cicadellidae genera in which chromosome number has been sufficiently liable to change in the course of speciation whereas most genera have a stable number of chromosomes (Kuznetsova and Aguin-Pombo in press). As mentioned above, *Alebra albostriella* and *Alebra wahlbergi* are the first representatives of the leafhopper genus *Alebra* studied in respect to karyotype. The chromosome complement of 2n = 22 + X(0) found in thesespecies is fairly common in the Cicadellidae (Kuznetsova and Aguin-Pombo in press) but has never been recorded for the subfamily Typhlocybinae in which approximately 90 species in 35 genera are presently known cytologically (Kirillova 1987, Aguin-Pombo et al. 2006, Juan 2011). The only exception is probably a species referred to as Gen. nov. 6 for which Juan (2011) recorded n=12 (suggesting thus 2n = 22 + X).

Cytological analysis of male meiosis in *Alebra albostriella* and *Alebra wahlbergi* revealed that it was of the typical auchenorrhynchan type where all the chromosomes undergo segregation at anaphase I and chromatids separate at anaphase II (Halkka 1959). The small bivalents invariably had only one terminal/subterminal or, rarely, interstitial chiasma. In the larger bivalents one-two chiasmata were formed; in separate diakinesis cells up to six ring bivalents with two terminal/subterminal chiasmata were present. Halkka (1964) was the first to show that only one-two chiasmata are typically formed in male meiosis in Auchenorrhyncha species. Quite recently, Nokkala et al. (2004) have proved that the low number of chiasmata (one-two in a bivalent from a cytological standpoint) is characteristic of holokinetic chromosomes as such. The authors showed that holokinetic bivalents with multiple chiasmata entered AI but were unable to complete it as a result of wrong separation of homologues. It is noteworthy however that in our material the largest bivalents showed occasionally three and even four chiasmata. Multiple (more than two) chiasmata have also been described in other groups with holokinetic chromosomes, including in the Auchenorrhyncha (e.g. Kuznetsova et al. 2009a, b, Maryańska-Nadachowska et al. 2012), however, the work by Nokkala et al. (2004) still remains the only one in which the further fate of cells with such bivalents in meiosis was traced in detail.

In the majority of auchenorrhynchans, at least in all hitherto studied planthopper species (Fulgoroidea), the univalent X-chromosome shows a clear tendency to be arranged at the periphery of MI plate presumably forming its own meiotic spindle (Halkka 1959, Kuznetsova et al. 1998, Maryańska-Nadachowska et al. 2006). In contrast to this, in both leafhopper species studied in the present work, the univalent X-chromosome tended to locate randomly among the bivalents at MI.

Polymorphism for chromosomal rearrangements, B-chromosomes and meiotic abnormalities are not rare in nature and has been recorded for numerous species of plants and animals, including insects (White 1973). This kind of information is however very scarce in the Auchenorrhyncha. In this group, among approximately 820 species studied cytogenetically, B-chromosomes and different chromosomal rearrangements have been reported in several species only (Kuznetsova and Aguin-Pombo in press). In light of this, it is somewhat unusual to find so extensive variation which occurs in *Alebra albostriella* and *Alebra wahlbergi* from Greece. A total of 62 individuals from 13 population samples belonging to both species were examined. The studied populations inhabited different food plants (*Fagus sylvatica*, *Castanea sativa*, *Quercus cerris*, *Acer opalus*) and different altitudes ranging from 20 m to 1200 m above sea level (Table 1). In all 6 populations of *Alebra albostriella*, 29 males (71%; N=41) showed meiotic abnormalities and of these 4 males displayed additionally B-chromosomes. In 5 populations of *Alebra wahlbergi*, 11 males (52%; N=21) showed meiotic abnormalities, and of these 2 males displayed also B-chromosomes. In addition, one further male showed B-chromosomes while no meiotic abnormalities. The remaining two populations of *Alebra wahlbergi*, each with the only studied specimen, showed neither B-chromosomes nor meiotic abnormalities.

### B-chromosomes

B-chromosomes are accessory genomic elements that are known to occur approximately in 15% of living species (Beukeboom 1994). In the Auchenorrhyncha, B-chromosomes were described in several species of planthoppers (Fulgoroidea) (Halkka 1959, Booij 1982, Kirillova and Kuznetsova 1990, den Bieman 1988) but have never been found to date in any species of leafhoppers (Cicadellidae). In leafhoppers *Alebra albostriella* and *Alebra wahlbergi* studied herein, B-chromosomes were found in low numbers (0-2) in 4 males of the first species (in 4 populations) and in 3 males of the second species (in 2 populations). In both species, B chromosomes were fairly small with the exception of male 1-AANE in which a single B-chromosome was about two times larger compared to Bs in other males (Fig. 2g). In every case however the Bs were much smaller than the univalent X-chromosome and conspicuous during meiotic prophase and metaphase I because of their negative heteropycnosis. When Bs were two in number, they did not pair and passed randomly through meiosis as univalents being still maintained in populations. Also, B-chromosome(s) did not connect to the univalent X-chromosome at MI as it was observed by Kirillova and Kuznetsova (1990) in several planthopper species from the family Delphacidae. The inter-population differences in B-chromosome distribution is believed to depend on different selective factors (Beukeboom 1994, Camacho 2004). It is interesting to note in this connection that in delphacid species *Javesella pellucida* (Fabricius, 1794) B-chromosomes were present only in populations inhabiting Northeast Siberia and Kamchatka whereas individuals sampled from different populations in European Russia lacked B-chromosomes (Kirillova and Kuznetsova 1990). In our study, the occurrence and frequency of B-chromosomes in males showed no relation with particular food plants on which populations inhabit. For example, *Alebra albostriella* males with Bs were collected both from *Castanea sativa* and *Fagus sylvatica*; *Alebra wahlbergi* males with Bs were sampled both from *Ulmus* sp. and *Acer opalus*. Similarly, no relationship was established between the occurrence of Bs and the habitat altitude above sea level: in *Alebra albostriella* Bs were present in populations inhabiting at 440 m and higher altitudes, while in *Alebra wahlbergi* they were found at lower altitudes (20m–520m).

In studiedpopulations, the frequency of B chromosomes was rather low. Thus, in *Alebra wahlbergi* they were present in 14% and in *Alebra albostriella* in only 10% of specimens studied. The frequency of individuals with 1B and 2Bs differed between the species. Thus, in four *Alebra albostriella* populations with B-chromosomes, 9,6% of males carried 1B and 3,2 % carried 2B, whereas in two *Alebra wahlbergi* populations with Bs, 11% of males had 1B and 22% had 2B. The data concerning the frequency of B chromosomes in natural populations of these species are too scarce to draw any firm conclusions. Noteworthy that the frequency of cells with B-chromosomes in 2B-males was markedly higher compared with that in 1B-carriers: 60,7%-83,7% against 0,65%-1,8% (Table 4). This observation suggests the existence of an accumulation mechanism responsible for maintaining the 2Bs in studied populations. Interesting, no males with more than two Bs were found in studied specimens. One can suppose that in *Alebra* populations, 1 or 2 B-chromosomes are tolerable to B-carriers and that natural selection operates by eliminating individuals with more than two Bs. In contrast, in the aforesaid planthopper species *Javesella pellucida*, males with up to four B-chromosomes were found (Kirillova and Kuznetsova 1990). However, in this species males with larger number of Bs were less frequent: males with 1B predominated (89%), males with 2Bs and 3Bs occurred with equal frequency (56%) whereas males with 4Bs were rare (11%).

The question of the adaptive significance of B-chromosomes in natural populations has been argued over for decades (Camacho et al. 2000), with the final position showing little if any substantial evidence to support such a role (Jones and Houben 2003). Among many others, the influence of B-chromosomes on recombination through the modulation of chiasma frequency and distribution in A-chromosomes has been recorded (e.g. in Orthoptera; Camacho et al. 2000). In *Alebra albostriella* and *Alebra wahlbergi*, intraindividual analysis demonstrated that chiasma frequency in a nucleus was independent of the occurrence and number of B-chromosomes that it contains. Similarly, there was no relationship between the occurrence of B-chromosomes and the occurrence, frequency and types of meiotic abnormalities in a male. As an example, B-chromosomes were absent in male 1-WNE which had bivalents with three chiasmata and in males 2, 3 and 4 of ASE with the highest percent of meiotic abnormalities (Tables 2 and 3). In some insects, B-carriers have shown a significant increase of the number of macrospermatids (Suja et al. 1986) however in our material such a correspondence was not observed.

### Meiotic abnormalities

As noted above, information on chromosome rearrangements and meiotic disturbances in auchenorrhynchan species is very scarce. A number of meiotic abnormalities including agmatoploidy (a result of fission of holokinetic chromosomes), aneuploidy, loose pairings of sex chromosomes and shrinkage of cytoplasm (changes in cytoplasmic volume) were described in three biotypes of the brown planthopper *Nilaparvata lugens* (Stål, 1854) from the family Delphacidae (Goh et al. 1992). A translocation polymorphism was encountered in the Australian leafhopper *Alodeltocephalus draba* Evans, 1966 (Whitten and Taylor 1969). Some other examples are presented in a review of Kuznetsova and Aguin-Pombo (in press). In *Alebra albostriella* and *Alebra wahlbergi*, one or another of meiotic abnormalities were found such as univalents, bivalent chains resulting from end-to-end non-homologous achiasmate associations, multiple translocation chains, anaphasic laggards and macrospermatids.

### End-to-end non-homologous associations

In males originating from different populations of *Alebra albostriella* and *Alebra wahlbergi*, end-to-end associations between bivalents were found at different stages of meiosis. The chains, involving up to four bivalents and occasionally (in *Alebra albostriella*) also the X-chromosome, were formed during prophase and were still intact at MI. In these cases, the persistent association was certainty non-chiasmate. Non-homologous telomeres did not touch intimately each other but unstained gaps were present between bivalents. Since in both species chromosomes display distal heterochromatic blocks (our unpublished data), one can suggest that the formation of artificial bivalent chains (pseudomultiples) is caused by heterochromatin adhesion due to which non-homologous chromosomes easily attract to one another.

Terminal associations of non-homologous bivalents without chiasma formation have been described in many plants and animals (White 1973, John and King 1982, 1985). These associations may involve from two up to all bivalents of a species. For instance, in the “holokinetic” moth species *Sphinx ligustri* (Linnaeus, 1758) (Lepidoptera, Sphingidae), all of the 28 bivalents were non-homologously attached to each other throughout prophase until prometaphase in females. Late in meiosis, the chains were broken down sequentially however short chains of two or three bivalents were still present at metaphase I. Like in the two *Alebra* species, in this moth non-homologous telomeres did not touch intimately each other but an unstained gap or some chromatin threads were seen between bivalents (Nokkala 1987).

### Translocation chains

**Figure 4.**
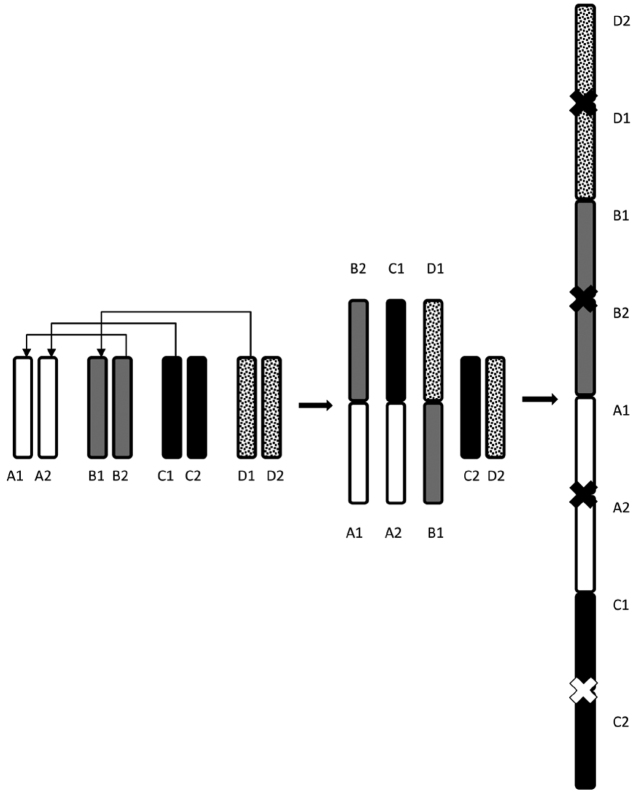
Schematic representation of the possible formation of a multiple translocation chain of four bivalents in meiosis of 1-WANE male. A1A2, B1B2, C1C2 and D1D2 are autosomal bivalents consecutively involved in translocation. Chiasmata in a translocation chain are shown by crosses.

In male 1-WANE sampled from *Castanea sativa*, part of nuclei had chains of several bivalents, non-homologous chromosomes showing the apparent intimate contacts by terminal chiasmata (Fig. 3g). In this male, the occurrence of heterozygotes for translocations could account for the multivalent chains formation. Since this chromosome rearrangement was observed only in some of meiotic cells, it must have happened after germinal cell development. Unfortunately, using only classical cytogenetic methods it was possible neither to affirm which chromosomes formed the chains nor to identify the orientation of separate chromosomes within a chain. Schematic representation of the possible formation of the chain-of-four caused by multiple translocations in meiotic cells is presented on Fig. 4.

In natural populations, chromosomal rearrangements arise as heterozygotes but their probability to establish is low (King 1993). Interchanges of small terminal segments of chromosomal pairs relatively frequently happen as spontaneous mutations (Hewitt 1979, Reed et al. 1992). Several studies have shown the chiasmate multivalent configurations, either rings or chains, in first meiosis as a result of heterozygosity for chromosomal fissions and fusions (e.g. in orthopterans; White 1973, John 1987, Bidau and Mirol 1988, Mirol and Bidau 1994, Colombo 2013). These configurations can lead to irregular segregation and nondisjunction in meiosis, with a consequent reduction in reproductive potential (White 1973), although sometimes they have no apparent negative influence on fertility (Mirol and Bidau 1994). Regarding insects with holokinetic chromosomes, the rearrangements can be even less deleterious since diffuse kinetochore is spreading through the length of their chromatids and products of rearrangements are able to be transmitted to daughter cells at successive cell divisions. Heterozygous translocations have occasionally been recorded in natural populations of “holokinetic” insects such as aphids (Blackman and Takada 1975, 1977, Blackman et al. 1995), heteropterans (Papeschi 1994, Bressa et al. 1998, Pérez et al. 2004) and psyllids (Grozeva and Maryańska-Nadachowska 1995, Nokkala et al. 2006). Within Auchenorrhyncha, a fascinating case of translocation polymorphism was described by Whitten and Taylor (1969) in populations of the Australian leafhopper species *Alodeltocephalus draba*. In all of the 8 studied populations, males showed one of the following chromosome complements: 3AA (three bivalents) + X; 4AA + X; 2AA + 1AAA (trivalent) + X; 1AA + 1AAAA (tetravalent) + X. A peculiar feature of this case is that the reduction in chromosome number has reached different stages in different localities. In one area (Lake Pedder), it was nearly or almost fixed (2 n= 7: 3AA + X), while in another area (Bruny Island) chromosome number decreased from north to south. Reduction of chromosome number following a latitudinal cline was caused by differences in the frequency of chromosome translocations. *Alodeltocephalus draba* was proposed to be under a process of speciation driven by the reorganization of karyotype which was initiated in some populations by the fixation of a particular chromosome fusion (Whitten and Taylor 1969).

### Univalents

In separate males of *Alebra albostriella* and *Alebra wahlbergi*, univalents of one-two chromosome pairs were observed at MI. The univalency involved either one of the larger or one of the smaller pairs of autosomes or occasionally both of these pairs. Although synaptic abnormalities can be responsible for the induction of abnormal chromosome segregation, no abnormal spermatids were observed in males showing univalents at metaphase I cells. This observation suggests a regular segregation of univalents in meiosis as it has been demonstrated by Nokkala (1986) for holokinetic univalents in the true bug species *Rhabdomiris striatellus* (Fabricius, 1794) (originally listed by Nokkala as *Calocoris quadripunctatus* (Vil.)) (Miridae, Hemiptera).

### Aberrant spermatids

Macrospermatids were encountered in males of both species. Aberrant spermatids occurred in small proportion in part of spermatocysts and were twice and sometimes several times as much as normal spermatids within the same cyst. In *Alebra albostriella*, abnormal spermatids were more abundant being found in four populations and in about 37% of the specimens studied. Chromosomal abnormalities that affect gametogenesis are known to be one of the principle causal factors in nonbalanced gametes appearance (White 1973). If the emergence of abnormal spermatids is a reflection of meiotic disturbances, one would expect a correspondence between the amount of macrospermatids and that of meiotic abnormalities in a male. Despite of this, there was a clear discrepancy between these two parameters. Macrospermatids instead of being abundant in males with numerous abnormalities were either occasional or absent and vice versa. For instance, in *Alebra albostriella*, out of 15 males displaying macrospermatids, 10 had no evident meiotic abnormalities at diakinesis and MI. Notice however that the fate of abnormal meiotic configurations was not traced in any of the individuals analyzed.

## Conclusion

It is not known at this stage what are the primary causes of abnormal chromosome behavior in males of *Alebra albostriella* and *Alebra wahlbergi* from Greek populations i.e. whether these causes are male-specific meiotic mutants, some environmental mutagens or the result of hybridization events between coexisting species on the same tree. Also it is not known whether these meiotic abnormalities may play a role in the karyotype evolution and speciation of the genus *Alebra*. This genus seems to be prone to chromosomal rearrangements that makes it an interesting group for further studies. From the cytological viewpoint, a greater number of species and samples as well as more detailed analysis employing special techniques like chromosome bandings and fluorescent *in situ* hybridization may help in determining the actual variety and frequency of chromosomal abnormalities and their contribution into the karyotype differentiation in the genus *Alebra*.
